# Maternal Misperception of Child Body Size and Its Association with Information-Seeking Opportunities and Information Sources in Japanese Preschool Children

**DOI:** 10.3390/children13030390

**Published:** 2026-03-11

**Authors:** Tomomi Kobayashi, Kemal Sasaki, Yuki Tada, Yasuyo Wada, Tetsuji Yokoyama

**Affiliations:** 1 Department of Food Science and Nutrition, Mukogawa Women’s University, Nishinomiya 663-8558, Japan; 2 Department of Food and Health Sciences, Jissen Women’s University, Tokyo 191-8510, Japan; sasaki-kemal@jissen.ac.jp; 3 Department of Nutritional Science, Tokyo University of Agriculture, Tokyo 156-8502, Japan; y3tada@nodai.ac.jp; 4 Department of Health Promotion, National Institute of Public Health, Wako 351-0197, Japan; wada.y.aa@niph.go.jp (Y.W.); yokoyama.t.aa@niph.go.jp (T.Y.)

**Keywords:** maternal misperception, child body size, preschool children, information-seeking, Maternal and Child Health Handbook, growth monitoring, Japan

## Abstract

**Highlights:**

**What are the main findings?**

- Maternal misperception of child body size, including both overestimation and underestimation, is prevalent among Japanese preschool children.
- Use of healthcare providers as an information source was independently associated with maternal overestimation of child body size.

**What are the implications of the main findings?**

- Contact with healthcare providers may reflect maternal concern regarding child growth and warrants further investigation in longitudinal studies.
- Support strategies that help caregivers interpret objective growth indicators are needed to reduce maternal misperceptions.

**Abstract:**

**Background/Objectives**: This study examined associations between maternal misperception and information-seeking opportunities, behaviors, and sources among Japanese mothers of preschool children. **Methods**: A cross-sectional online survey was conducted among mothers registered with a nationwide research panel. Mothers of children aged 3–5 years were included because, in Japan, this period follows the last early-childhood health checkup at age 3, after which caregivers are required to monitor child growth independently. In total, 1358 mothers were analyzed. Child anthropometric data were reported by mothers with reference to the Maternal and Child Health Handbook or childcare records. These measurements were originally obtained during routine health checkups conducted by healthcare professionals or childcare staff. Body mass index z-scores were categorized as high, middle, or low, and maternal perception as accurate, overestimated, or underestimated. Information-seeking behaviors were assessed using study-specific items informed by prior literature and reviewed by experts to ensure content and face validity. Health literacy was measured using the validated 12-item Japanese Health Literacy Scale, which has demonstrated reliability and validity in previous studies. Multinomial logistic regression was used. **Results**: Among children with high body size, 150/188 (80.8%) of mothers underestimated body size; among those with low body size, 20/35 (57.1%) overestimated it. In multivariable analyses, use of healthcare providers as an information source was statistically associated with maternal overestimation of child body size. **Conclusions**: Maternal misperception was common across body size categories. Further research is needed to determine whether support in interpreting objective growth indicators is associated with improved perception accuracy.

## 1. Introduction

An imbalanced nutritional status during childhood, including both underweight and overweight, is a major public health concern. In particular, the preschool period is a critical stage for growth and development, during which health-related behaviors are established. Body size and nutritional status during this period may influence health outcomes across the life course [1]. Young children’s lifestyle behaviors are largely shaped by the home environment, and caregivers play a central role in managing children’s eating behaviors, physical activity, and overall health. Therefore, maternal perception of the child body size is considered an important factor influencing daily caregiving practices and health-related responses. Recent studies and systematic reviews continue to document substantial discrepancies between caregivers’ perceptions and objectively assessed child body size [2,3,4,5,6,7], underscoring that maternal misperception remains a persistent public health concern. In settings where higher body weight has become increasingly prevalent, a shift in normative standards may contribute to the social normalization of larger body sizes, making excess weight less likely to be recognized as problematic. Recent studies suggest that parents may perceive larger body sizes as typical or healthy within their social environment, thereby contributing to the underestimation of overweight in children [2,5]. Importantly, parents’ misperceptions about their child’s physique can be considered an important upstream factor shaping subsequent dietary habits and healthcare-seeking behavior. Both underestimating and overestimating a child’s physique can lead to inappropriate responses. For example, underestimation may delay recognition of malnutrition or growth stunting, while overestimation risks prompting unnecessary dietary restrictions or excessive interventions. Therefore, parents’ accurate perception of their child’s physique is not merely an outcome of concern but also a crucial starting point for appropriate pediatric health management.

Parental perception of child body size is influenced not only by personal values and sociocultural ideals, but also by access to health-related information and the ability to understand and use such information. In Japan, under the Maternal and Child Health Act, all pregnant women are issued the Maternal and Child Health Handbook (MCHH), and standardized growth monitoring using growth charts is implemented nationwide through routine health checkups for infants and young children [8]. Guaranteed exposure to standardized growth criteria from birth is a distinctive feature of Japan’s maternal and child health system. Notably, the MCHH enables caregivers to monitor their own child’s growth longitudinally using standardized growth charts, allowing evaluation based on changes over time rather than comparisons with other children. This self-referenced, longitudinal growth monitoring may function as a corrective mechanism for parental perception, helping caregivers align their subjective impressions with objective growth indicators. However, whether such theoretically favorable systems actually function as intended in real-world settings remains uncertain. Meanwhile, in recent years, the range of health information sources available to caregivers has expanded, including the internet, social media, and childcare-related applications. Although these digital information sources offer high accessibility, many are not supervised by healthcare professionals, resulting in substantial variability in accuracy and reliability, and the widespread dissemination of information that is not necessarily evidence-based [9]. Low health literacy (HL), defined as the ability to obtain, understand, and use appropriate health information, has been associated with inappropriate health behaviors and distorted health perceptions [10,11,12]. Accordingly, caregivers’ levels of HL may influence their understanding and perception of their child body size [13,14,15]. However, few studies have directly examined whether general HL is associated with caregivers’ perception of child body size.

Many previous studies examining the relationship between maternal perception of child body size and opportunities or information sources regarding appropriate body size have been conducted in countries or regions where growth monitoring opportunities and information provision systems are limited [16,17,18,19]. However, even in social environments such as Japan, where standardized growth monitoring systems exist, it remains unclear whether mere access to such systems actually translates into accurate parental perception. Moreover, even in such environments, the factors associated with discordance in maternal perception of child body size remain insufficiently understood. Although such systems are theoretically expected to support accurate perception, empirical evidence on their actual effectiveness remains limited. Few studies have systematically examined how opportunities to obtain information and the types of information sources used by mothers influence the accuracy of body size perception.

From a theoretical perspective, the relationship between information use and perception accuracy may operate through several mechanisms. Firstly, exposure to standardized growth indicators may facilitate cognitive recalibration of subjective judgment by aligning caregivers’ impressions with objective growth references (cognitive mechanism). Secondly, active information-seeking behaviors may reflect greater engagement in child health monitoring and decision-making processes, thereby promoting more attentive evaluation of the child body size (behavioral mechanism) [20]. Thirdly, the credibility, framing, and professional supervision of information sources may shape how caregivers interpret and internalize growth-related information (informational mechanism) [21,22]. Despite these theoretical pathways, empirical evidence directly examining these mechanisms remains limited.

Therefore, this study examined the association between discordance in maternal perception of child body size and mothers’ information-seeking opportunities, information-seeking behaviors, and information sources related to appropriate body size among Japanese mothers of preschool children.

## 2. Materials and Methods

### 2.1. Study Design and Participants

An online cross-sectional survey was conducted in January 2024. An online research company (NTT Com Online Marketing Solutions Corporation, Shinagawa, Tokyo, Japan) invited 1400 mothers registered on their online panel to participate in the survey. Eligible participants were mothers residing in Japan whose native language was Japanese and who lived with a child aged three to five years. The exclusion criteria included mothers of multiple births and those working as healthcare professionals (physicians, dentists, pharmacists, nurses, public health nurses, midwives, clinical laboratory technicians, dietitians, registered dietitians, physical therapists, occupational therapists, or speech–language–hearing therapists). Mothers of multiple births were excluded to maintain comparability within the study population, as twins and higher-order multiples are known to have distinct growth trajectories and caregiving dynamics that may influence both objectively assessed body size and maternal perception. Based on an expected prevalence of maternal misperception (overestimation plus underestimation) of approximately 30%, we aimed to recruit approximately 1400 participants to estimate this prevalence with reasonable precision, corresponding to a 95% confidence interval half-width of approximately 2–3 percentage points. This estimate was informed by a prior independent pilot survey conducted in a separate sample using a similar questionnaire framework (unpublished data). Recruitment feasibility within the online survey panel was also considered when determining the target sample size. Participants were instructed to enter their child’s anthropometric data while referring to available records, such as the Maternal and Child Health Handbook or childcare records (e.g., nursery school or kindergarten health logs), to improve reporting accuracy. The Maternal and Child Health Handbook contains anthropometric measurements recorded by healthcare professionals during routine health checkups. Although the measurements were not independently verified, biologically implausible child BMI percentiles were excluded to enhance data quality. Because anthropometric data were self-reported by mothers, some degree of reporting bias or misclassification cannot be excluded. However, as mothers were instructed to refer to the MCHH, any misclassification is likely to be non-differential with respect to perception status.

**Figure 1:**
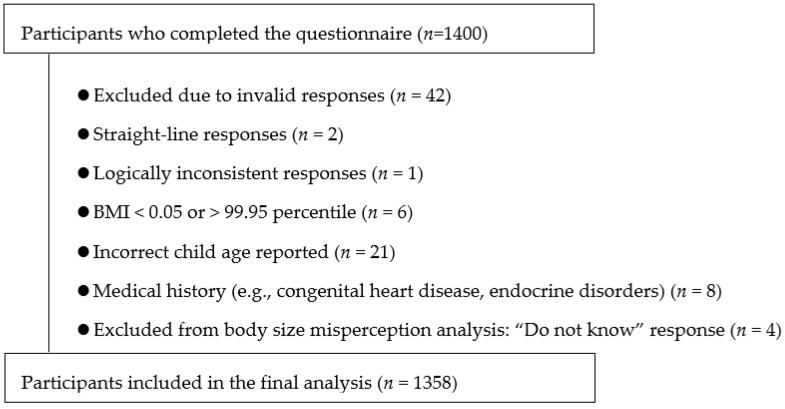
Flowchart of participant selection.

To ensure data quality, responses deemed invalid based on predefined criteria were excluded from analysis. Specifically, the exclusion criteria were straight-line responses to questionnaire items (*n* = 2), logically inconsistent responses (*n* = 1), extreme child BMI percentiles (<0.05 or >99.95 percentile) at the time of the survey (*n* = 6), incorrect reporting of the child’s age (*n* = 21), reported medical histories that could affect growth (e.g., congenital heart disease or endocrine disorders; *n* = 8), and respondents who reported that they did not know their child body size (*n* = 4). The child BMI percentile values were calculated based on spline-smoothed reference data rather than assuming a normal distribution. Therefore, the number of excluded cases may not necessarily correspond to the theoretically expected number under a normal distribution. After these exclusions, data from 1358 participants were included in the final analysis (Figure 1).

### 2.2. Survey Measures

Information was collected on maternal (age, employment status, highest educational attainment, self-reported height and weight, and HL) and child (gestational age, age, sex, daytime childcare attendance, household composition, height and weight at birth and at the time of the survey, food allergies, and medical history) characteristics. Maternal perception of child body size, information-seeking behaviors related to appropriate body size, opportunities to learn about appropriate body size, and information sources regarding appropriate body size were also assessed.

Information-seeking behavior was assessed using study-specific questionnaire items developed with reference to previous literature on parental health information seeking [20]. The content and wording of the instrument were reviewed by the research team, including experts in public health and maternal and child health, to ensure content and face validity. Because the instrument was newly developed for this study, formal psychometric validation, including assessment of reliability and construct validity, was not conducted. Self-initiated information seeking was defined as active searching for information regarding appropriate child weight or height, whereas opportunity to learn was defined as passive exposure to information regarding appropriate child body size. Each information source variable was coded as a binary indicator (used/not used); frequency or intensity of use was not assessed. All information-seeking variables referred to behaviors occurring prior to or concurrent with the assessment of maternal perception.

Participants were asked to select all applicable sources from which they had obtained information about appropriate child body size from a predefined list of 16 sources. These variables represent mothers’ reported sources of information, not survey participants. For analysis, these sources were grouped into five broader categories based on the type of provider or medium: (1) public health professionals (including physicians, dietitians/nutritionists, nurses, midwives, and dental staff in medical institutions and public health centers), (2) childcare professionals (including dietitians, teachers, nurses, and childcare providers in preschool settings), (3) family or friends, (4) internet and social media (including SNS, websites, mobile applications, and online platforms), and (5) the Maternal and Child Health Handbook (MCHH). Each category was coded as a binary variable and considered “used” if the participant reported using at least one source within that group. Mothers were asked to enter their child’s height and weight at birth, at the 3-to 4-month health checkup, at the 18-month health checkup, at the 3-year health checkup, and at the time of the survey while referring to the MCHH.

### 2.3. Statistical Analysis

BMI z-scores were computed based on Japanese reference data [23]. Body size categories were defined according to the methodology proposed by Shinoda et al. [24] for Japanese preschool children. In this approach, a BMI z-score ≥+1 corresponds to the WHO criterion for “possible risk of overweight”, and a z-score <−2 corresponds to underweight. Maternal BMI was calculated using self-reported height and weight and included as a covariate in the multivariable models as a potential confounder, because maternal BMI may be associated with both child body size and maternal perception [25].

Maternal perception of child body size was assessed using subjective categories (“high”, “medium”, or “low”) and classified as accurate, overestimated, or underestimated based on agreement with objectively defined body size categories. In the high body size group, the perception of being “overweight” was considered accurate, and all other responses were classified as underestimated. In the middle group, the perception of “normal” was considered accurate, “overweight” as overestimated, and “underweight” as underestimated. In the low group, the perception of “underweight” was considered accurate, and all other responses were classified as overestimated. In this study, both overestimation and underestimation were defined as maternal misperceptions of the child body size.

HL was assessed using the Japanese version of the 12-item Health Literacy Scale (HLS-Q12) [26]. Responses were rated on a four-point Likert scale (1 = very easy to 4 = very difficult). HL index scores were standardized to a scale ranging from 0 to 50 using the following formula: index = (mean score − 1) × (50/3) [27].

Categorical variables were examined using Fisher’s exact test. A multinomial logistic regression analysis was conducted to identify factors associated with maternal misperception of the child body size, with accurate perception as the reference category. Results are presented as adjusted odds ratios (ORs) with 95% confidence intervals (CIs). Two hierarchical models were constructed. Model 1 was adjusted for child-related factors: child’s age at the time of the survey (continuous), sex (reference: girl), childcare attendance (reference: none), and gestational age (continuous). Model 2 was additionally adjusted for maternal factors: maternal age (continuous), maternal BMI (continuous), employment status (reference: unemployed), and HL score (continuous). For analyses examining associations between maternal misperception of child body size and information sources, mothers’ information-seeking behaviors related to child growth (reference: none) were additionally adjusted in Model 2. All variables were simultaneously entered into each model. Multicollinearity was assessed by calculating variance inflation factors (VIFs) using a linear regression model with the same independent variables as those included in the multinomial logistic regression. All VIFs were close to 1.0 (range: 1.006–1.189), indicating no evidence of problematic multicollinearity.

All statistical tests were two-sided, and *p*-values < 0.05 were considered statistically significant. Statistical analyses were performed using the IBM SPSS Statistics (version 30.0; IBM Corp., Armonk, NY, USA).

### 2.4. Ethical Considerations

This study was conducted in accordance with the principles of the Declaration of Helsinki and approved by the Ethics Committee of Jissen Women’s University (approval number: H2023-29; approval date: 11 January 2024). Informed consent was obtained from all participants prior to participation. Data were collected anonymously, and participants were informed that their responses would be used solely for research purposes.

## 3. Results

### 3.1. Participant Characteristics

Table 1 shows the maternal perception of child body size according to the objectively defined body size categories. More than half of the mothers of children classified into either the high or low body size groups misperceived their own child body size.

Table 2 shows the characteristics of the study participants. No significant differences were observed among the three groups in terms of agreement between maternal perception and objectively defined body size with respect to child age, gestational age at birth, maternal age, attendance at nursery school or kindergarten, maternal BMI, HL score, or employment status.

### 3.2. Association Between Maternal Misperception of Child Body Size and Information-Seeking Behavior and Information Sources on Appropriate Body Size

Table 3 shows the associations between maternal misperception of child body size and information-seeking behaviors as well as information sources related to appropriate body size. Significant associations were observed between maternal misperception of child body size and self-initiated information-seeking behaviors regarding appropriate child weight and height, as well as the use of healthcare professionals as information sources in the univariate analyses. Compared with the accurate perception group, the proportion of mothers who did not engage in information-seeking behaviors and those who did not use healthcare professionals was significantly higher in both the overestimation and underestimation groups. In contrast, no significant associations were observed between maternal misperception of child body size and the use of public health professionals, childcare and early childhood education professionals, family or friends, the Internet or social media, or the MCHH.

Table 4 presents the results of the multinomial logistic regression analysis. Factors independently associated with maternal misperception of child body size differed between the overestimation and underestimation groups in the multivariate model. In the overestimation group, mothers who engaged in self-initiated information seeking regarding appropriate child weight and height and those who used healthcare providers as information sources were more likely to overestimate their child body size. The fully adjusted multinomial logistic regression model (Model 2) showed a significantly improved fit compared with the intercept-only model (LR χ^2^ (18) = 42.141, *p* = 0.001). The Nagelkerke R^2^ was 0.040, indicating limited explanatory power.

In contrast, no statistically significant associations were observed between information-seeking behaviors or information sources and the underestimation of child body size. For example, the association between lack of self-initiated information seeking and underestimation did not reach statistical significance (OR 0.79, 95% CI 0.62–1.02), and the confidence interval included unity, suggesting estimation uncertainty.

Detailed results for the adjustment variables are presented in Supplementary Tables S1 and S2. Among the covariates included in the multivariate analysis, child sex was associated with maternal misperception of child body size; male sex was associated with a lower likelihood of overestimation and a higher likelihood of underestimation than female sex. In addition, younger age and attendance at nursery school or kindergarten were associated with an underestimation of child body size. No significant associations were observed between maternal misperception of child body size and maternal age, gestational age at birth, maternal BMI, HL scores, or maternal employment status. Furthermore, additional adjustments for birth weight z-scores did not materially change the direction or significance of the main associations.

## 4. Discussion

This study aimed to examine the associations between maternal misperception of child body size and mothers’ information-seeking opportunities, information-seeking behaviors, and information sources related to appropriate body size among Japanese preschool children.

The prevalence of underestimation observed in this study (80.8%) is situated at the upper end of values reported internationally. In the WHO European Childhood Obesity Surveillance Initiative (COSI), conducted across 22 countries, the proportion of parents who underestimated their child’s weight status ranged from 53% to 90% [2]. Similarly, a study of preschool children in China reported that many parents did not accurately recognize their child’s weight status [3], and a study from New Zealand also documented discrepancies between caregiver perceptions and objectively measured weight status among young children [4]. Furthermore, a recent systematic review and meta-analysis demonstrated that parental underestimation of overweight among children and adolescents remains widespread across different countries and age groups [5]. Compared with these international findings, the prevalence observed in the present study lies near the upper bound of previously reported estimates. One possible explanation for this relatively high prevalence is the difference in population-level BMI distributions. The average BMI of Japanese children is lower than that reported in many Western populations [28]. As a result, children with relatively higher BMI values may still appear “normal” when compared with their peers in the immediate social environment. Previous studies have also reported that parental underestimation of child overweight is common and may be influenced by parental beliefs and perceptions regarding body size [25,29]. Previous research conducted in Japan among school-aged children has also suggested that parental perceptions of childhood obesity are associated with children’s weight status. For example, a Japanese population-based study reported that lower parental recognition of childhood obesity was related to a higher likelihood of overweight among children, highlighting the potential importance of parental awareness in childhood obesity prevention [30]. Although such research remains limited in Japan, these findings suggest that parental perception may play an important role in early identification of unhealthy weight status.

In contrast, the overestimation observed among mothers of children in the low body-size group has been less frequently examined internationally. However, previous studies suggest that parental perception of child weight status may be influenced by parental beliefs and sociocultural attitudes toward body size [26]. In Japan, thinness-oriented body ideals are particularly prevalent among young women [31,32,33,34]. Such sociocultural norms may contribute to the relatively high proportion of overestimation observed among mothers of children in the low body-size group in the present study. Taken together, these findings suggest that maternal perception of child body size is shaped not only by objective growth indicators but also by broader sociocultural contexts and body image norms.

The cutoff values used to classify child body size in this study (BMI z-score ≥+1 and <−2) were based on the methodology proposed by Shinoda et al., which was developed for Japanese preschool children [24]. Shinoda et al. indicated that a BMI z-score of +1 corresponds to the WHO criterion for “possible risk of overweight”. By adopting this threshold, which is more sensitive than the clinical definition of obesity (commonly defined as ≥+2 SD), this study was able to assess how mothers perceive their children’s relative body size, rather than focusing on clinical diagnosis. Similarly, defining a BMI z-score <−2 as the low body size group was consistent with the medical criteria for underweight and was considered an appropriate indicator for evaluating maternal perception of thinness. The primary aim of this study was not to identify clinically overweight or obese children but to examine discordance between maternal subjective perception and objectively defined relative body size. Therefore, adopting a more sensitive threshold (≥+1 SD), corresponding to “possible risk of overweight”, was considered appropriate to capture early perceptual discrepancies. Using the conventional obesity cutoff (≥+2 SD) would have resulted in a substantially smaller high body size group, thereby limiting the ability to examine perceptual discordance in its early stages. The term “high” in this study does not indicate a clinical diagnosis of overweight or obesity but rather reflects a relative position within the population-specific BMI distribution.

A substantial proportion of mothers of children classified in the high body size group underestimated their own child body size (80.8%). This finding is consistent with multinational studies and systematic reviews reporting a persistent tendency for parents to underestimate overweight or obesity across diverse cultural contexts [2,3,5]. For example, a Dutch study reported that 64.7% of parents of overweight children underestimated their child’s weight status in 2009, and 61.0% did so in 2013; underestimation was even more pronounced among parents of obese children (95.5% in 2009 and 93.0% in 2013) [29]. The prevalence observed in the present study falls within the range reported in previous European studies, suggesting that such misperception is not limited to a specific sociocultural context. In addition, this study revealed that approximately half of the mothers of children in the low body-size group overestimated their child body size. While much of the existing literature has focused on the underestimation of overweight, fewer studies have examined parental overestimation of thinness, particularly in East Asian settings. In Japan, the proportion of women with a BMI < 18.5 kg/m^2^ is higher among those in their twenties than among those aged 30–60 years [31]. Previous studies have also reported that thinness-oriented body ideals are prevalent among young Japanese women and that some women perceive themselves as overweight even when they are underweight or of normal weight [32,33,34]. Given that the BMI distribution of Japanese women is relatively low compared with other countries [35], maternal body image norms may be associated with maternal evaluations of child body size; however, this relationship was not directly assessed in the present study. This sociocultural context may be one possible factor underlying the relatively high proportion of overestimation observed among mothers of children with low body size.

In the underestimation group, mothers of boys were significantly more likely to underestimate their child body size than mothers of girls, consistent with findings in school-aged children [36], suggesting that gender-related expectations regarding body size may emerge early in childhood. Similar sex differences in parental perception have been reported in recent studies [25], suggesting that sociocultural norms associating larger body size in boys with strength or healthy growth may contribute to this pattern. However, this study did not directly assess maternal values such as mothers’ own body image, body ideals, or beliefs regarding child growth. Therefore, the psychological mechanisms underlying maternal misperception cannot be fully elucidated, and further research incorporating maternal values, sociocultural norms, and gender-related expectations is warranted. International studies have reported that failure to recognize thinness in children is associated with adverse health outcomes, including delayed growth and development, impaired immune function, and increased susceptibility to infections [37,38]. When caregivers do not perceive thinness as problematic, opportunities for timely nutritional support may be missed [37]. Previous reviews on weight communication have also highlighted that caregiver understanding and interpretation strongly influence responses to growth-related information [39]. While global research has predominantly focused on parental underestimation of overweight, comparatively less attention has been paid to the misperception of thinness, particularly in high-income countries where obesity prevention has been prioritized. In Japan, where public health messaging has historically emphasized the prevention of excessive weight gain, maternal misperception among mothers of underweight preschool children may represent an underrecognized issue. Future studies should clarify the determinants of discordance between objectively defined body size and maternal perception and explore preventive support strategies and effective communication approaches from a life-course perspective.

In the multivariable analysis, use of healthcare providers as an information source was statistically associated with maternal overestimation of child body size. Specifically, mothers who did not use healthcare providers had lower odds of overestimation compared with those who used such providers. These findings indicate that healthcare provider use and overestimation co-occurred within this study sample. However, given the cross-sectional design, the temporal sequence and directionality of this association cannot be determined. It is possible that consultation with healthcare providers influenced maternal perception; alternatively, pre-existing maternal concern regarding the child’s growth may have prompted healthcare utilization. Because routine check-ups may occur after maternal perceptions have already formed, healthcare utilization should be interpreted as a correlate of perception rather than evidence of a causal or corrective effect. Thus, healthcare use may reflect prior uncertainty or concern rather than a determinant of perception accuracy.

Although healthcare providers offer objective, standardized growth assessments during routine check-ups and provide feedback based on growth references, such consultations may occur after maternal perceptions have already formed. Therefore, healthcare utilization may represent an indicator of existing uncertainty or concern rather than a determinant of perception accuracy.

Previous evidence suggests that parental misperception of child weight status remains common, particularly among parents of younger children, and that communication from healthcare professionals can influence parental recognition and engagement with weight-related guidance [29]. However, given the cross-sectional design of this study, the temporal sequence between healthcare utilization and maternal perception cannot be determined. These findings should therefore be interpreted as associative rather than causal.

In contrast, no information-seeking behaviors or information sources were independently associated with the underestimation of child body size. A systematic review found that parental underestimation of overweight and obesity in children and adolescents is widespread across settings and age groups, highlighting the challenges of improving perception even when information is available [5]. In routine child health checkups and preventive guidance, greater emphasis is typically placed on concerns related to excessive weight gain and obesity prevention than on the potential risks associated with thinness in early childhood. Consequently, caregivers are more likely to receive explicit feedback regarding excessive weight gain than regarding low body size. This difference in emphasis may partly account for the finding that healthcare provider use appeared to be associated with overestimation but not with underestimation in the present study. Even when objective information is available, maternal underestimation may not be fully explained by differences in information access alone and may involve other unmeasured psychological or sociocultural factors. For children with a higher body size, caregivers may perceive their child’s size as “within the normal range” due to social normalization of larger body sizes or a positive bias toward growth and vitality. Such perceptions can persist despite the availability of objective growth information, suggesting that information-based approaches alone may be insufficient. Personalized feedback from healthcare professionals may therefore be required to address maternal underestimation.

Although the MCHH is a core component of Japan’s maternal and child health system [8], its use was not significantly associated with maternal perception accuracy in this study. Although a potential trend toward both overestimation and underestimation was observed among non-users, these associations did not reach statistical significance. One possible explanation is that the possession or use of the MCHH does not necessarily translate into an accurate interpretation of its growth information. Although the MCHH provides standardized growth charts, supporting accurate perception may depend not only on access to growth charts but also on caregivers’ ability to appropriately interpret the information provided. This interpretation is consistent with previous research indicating that the effectiveness of growth monitoring and weight communication depends not merely on the availability of growth data but also on how such information is explained and contextualized by health professionals [18,39]. Systematic reviews have suggested that communication about child weight status is complex and that caregivers’ understanding and responses are strongly influenced by the clarity and framing of professional feedback [39]. Therefore, the effectiveness of the MCHH may depend on whether caregivers receive an adequate explanation or support in interpreting the information it provides. Accordingly, findings related to specific information sources should be interpreted as exploratory and hypothesis-generating, given the observational design and the potential for multiple comparisons.

In this study, HL, as measured by a general HL scale, was not independently associated with maternal misperception of child body size. The HLS-Q12 used in this study is a short form derived from the HLS-EU-Q47 and shares the same theoretical framework; however, direct numerical comparisons between different versions of the instrument should be interpreted cautiously. Nevertheless, previously reported HLS-EU-Q47 scores from Japanese population-based studies provide a useful benchmark for interpreting the overall HL level of our sample [26]. The mean HL score observed in this study (27.8) was broadly comparable to those reported in Japanese adult samples, suggesting that the overall HL level of our participants did not markedly differ from prior studies. Recent studies have also highlighted that general eHealth or comprehensive HL does not always directly translate into accurate interpretation of specific clinical indicators, suggesting the need for more context-specific literacy measures [11]. Future studies may benefit from using growth- or nutrition-specific literacy measures to elucidate the role of caregivers’ interpretive skills in shaping body size perception.

Taken together, these findings indicate that simply increasing access to information may be insufficient to improve maternal perception of child body size. Rather, our results suggest that how growth-related information is communicated and interpreted—particularly within interactions with healthcare professionals—may be associated with patterns of maternal perception, although directionality cannot be inferred from this cross-sectional design. This interpretation is consistent with reports indicating that communication about a child’s weight is complex and that caregivers’ understanding and responses are influenced by the clarity, framing, and tailoring of professional feedback [40,41]. In Japan, where standardized growth monitoring systems and the MCHH are already widely implemented, strengthening professional explanations and interpretive support within existing healthcare and public health services may represent a potentially promising approach.

This study extends previous research by examining maternal misperception of child body size in relation not only to objective anthropometric indicators but also to mothers’ information-seeking opportunities, behaviors, and information sources.

This study has several limitations. Firstly, owing to its cross-sectional design, causal relationships between maternal perceptions and information sources cannot be determined. Longitudinal studies are needed to examine changes in maternal perception in relation to children’s growth trajectories and dietary patterns. Secondly, all data were collected through maternal self-report in a single online survey and may therefore be subject to recall bias and social desirability bias. Mothers were instructed to enter their children’s anthropometric data while referring to available records, such as the MCHH or childcare records, which typically contain measurements recorded by healthcare professionals or childcare providers. The MCHH contains anthropometric measurements recorded by healthcare professionals during routine health checkups. However, these measurements were not independently verified by the research team, and reporting errors or misclassification of body size categories cannot be ruled out. Because the anthropometric data were not measured specifically for this study, discrepancies in measurement timing or transcription errors may have occurred, and some mothers may have entered earlier rather than the most recent values. Such non-differential misclassification could have attenuated the observed associations. Moreover, the use of specific BMI z-score thresholds may have influenced classification boundaries and should be considered when interpreting patterns of misperception. As described in the Methods section, extreme BMI percentile values were excluded to improve data quality. Thirdly, because mothers commonly use multiple information sources, each source was examined in separate models to reduce multicollinearity and facilitate interpretation. Nevertheless, evaluating multiple sources increases the likelihood of chance findings due to multiple comparisons; accordingly, these results should be interpreted cautiously and confirmed in independent samples. The overestimation group was relatively small (*n* = 41), which may have affected the statistical stability of the multinomial logistic regression models. Given the limited number of outcome events relative to the number of covariates, overfitting or unstable parameter estimates cannot be excluded. Findings related to overestimation should therefore be interpreted with caution. Although the direction of associations was generally consistent with univariate analyses, the precision of the estimates was limited. Future studies with larger samples or alternative modeling approaches, such as penalized regression techniques, are warranted to provide more robust estimates. The modest Nagelkerke R^2^ values also suggest that additional unmeasured factors may contribute to maternal misperception. Fourthly, maternal values related to body image and beliefs about child growth were not directly assessed, limiting the ability to fully explain the psychological mechanisms underlying maternal misperception. Finally, participants were recruited from an online survey panel and may have been more familiar with ICT devices than the general population, which could have led to overestimation of information-seeking behaviors and information source utilization. Caution is therefore warranted when generalizing these findings to the broader population.

## 5. Conclusions

This study demonstrated that maternal misperception of child body size is common among Japanese preschool children across all objectively defined body size categories. Use of healthcare providers as an information source was associated with maternal overestimation within this study sample.

These findings indicate an association between healthcare provider use and maternal perception within this sample; however, the directionality of this relationship cannot be determined due to the cross-sectional design. Healthcare provider use may reflect prior parental concern rather than a protective effect. The results suggest that improving maternal perception may require more than simply increasing access to information. Supporting caregivers in understanding and applying objective growth indicators within routine healthcare settings may be beneficial, although the observed effect size was relatively small. Further longitudinal research is needed to clarify the directionality and underlying mechanisms of these associations.

## Figures and Tables

**Table 1:** Maternal perception of child body size by BMI category.

		Total (*n* = 1358)		Body Size Category ^2^						
				High (*n* = 188)		Medium (*n* = 1135)		Low (*n* = 35)		*p* Value ^1^
		*n*	%	*n*	%	*n*	%	*n*	%	
Weight perception	Overweight	58	4.3	36	19.1	21	1.9	1	2.9	<0.001
	Approximately the right weight	1051	77.4	146	77.7	886	78.1	19	54.3	
	Underweight	249	18.3	6	3.2	228	20.1	15	42.9	

^1^ *p* values were calculated using Fisher’s exact test. ^2^ Child body size was classified into three groups using sex- and age-specific BMI z-scores: high (≥+1 SD), middle (≥−2 SD to <+1 SD), and low (<−2 SD). BMI z-scores were calculated based on the Japanese reference data.

**Table 2:** Characteristics of participants by maternal misperception of child body size.

		Total (*n* = 1358)		Maternal Misperception of Child Body Size ^1^					
				Overestimation (*n* = 41)		Accurate (*n* = 937)		Underestimation (*n* = 380)	
		Mean	SD	Mean	SD	Mean	SD	Mean	SD
**Child characteristics**									
Age of child at survey (years)		4.2	0.8	4.3	0.7	4.2	0.8	4.1	0.8
Sex	Boy	668	(49.2)	13	(31.7)	443	(47.3)	212	(55.8)
	Girl	690	(50.8)	28	(68.3)	494	(52.7)	168	(44.2)
Gestational age (days)		276.3	8.1	275.8	7.1	276.3	8.0	276.4	8.5
Birth height (z score)		−0.007	1.063	−0.118	1.419	0.024	1.027	−0.073	1.104
Birth weight (z score)		0.123	0.874	0.229	0.802	0.158	0.860	0.026	0.910
BMI at survey(z score)		0.036	0.974	−0.960	1.750	0.030	0.795	0.156	1.180
Body size category ^1^	High	188	(13.8)	0	(0.0)	36	(3.8)	152	(40.0)
	Medium	1135	(83.6)	21	(51.2)	886	(94.6)	228	(60.0)
	Low	35	(2.6)	20	(48.8)	15	(1.6)	0	(0.0)
Attendance at nursery school or kindergarten	Attends	1270	(93.5)	36	(87.8)	873	(93.2)	361	(95.0)
	Does not attend	88	(6.5)	5	(12.2)	64	(6.8)	19	(5.0)
Maternal characteristics									
Maternal age (years)		34.6	5.0	35.0	5.3	34.7	4.9	34.3	5.1
Maternal body mass index (kg/m^2^)		21.1	3.3	21.1	3.2	21.1	3.3	21.0	3.4
Maternal health literacy (score of HLS-Q12)		27.8	8.3	30.2	7.0	27.8	8.3	27.7	8.3
Maternal educational attainment	≤High school	338	(24.9)	4	(9.8)	252	(26.9)	82	(21.6)
	≥Junior college	1020	(75.1)	37	(90.2)	685	(73.1)	298	(78.4)
Maternal employment status	Employed	853	(62.9)	27	(65.9)	558	(61.5)	238	(62.6)
	Not employed	503	(37.1)	14	(34.1)	349	(38.5)	142	(37.4)

^1^ Maternal perception of child body size (“overweight”, “normal”, or “underweight”) was compared with the child’s objectively defined body size category. Based on this comparison, maternal perceptions were categorized into three groups: overestimation, accurate perception, and underestimation.

**Table 3:** Information-seeking behaviors and sources related to appropriate body size according to maternal misperception of child body size.

		Total (*n* = 1358)		Maternal Misperception of Child Body Size ^2^						
				Overestimation (*n* = 41)		Accurate (*n* = 937)		Underestimation (*n* = 380)		*p* Value ^1^
		*n*	%	*n*	%	*n*	%	*n*	%	
Information Seeking Behavior										
Self-initiated information seeking about appropriate child weight/height	None	508	37.4	8	19.5	371	39.6	129	33.9	0.009
	Yes	850	62.6	33	80.5	566	60.4	251	66.1	
Opportunity to learn about appropriate weight/height	None	95	7.0	0	0.0	67	7.2	28	7.4	0.206
	Yes	1263	93.0	41	100.0	870	92.8	352	92.6	
Sources of information										
Healthcare providers	Not used	1023	75.3	22	53.7	722	77.1	279	73.4	0.003
	Used	335	24.7	19	46.3	215	22.9	101	26.6	
Public health professionals	Not used	883	65.0	25	61.0	605	64.6	253	66.6	0.669
	Used	475	35.0	16	39.0	332	35.4	127	33.4	
Childcare professionals	Not used	1198	88.2	39	95.1	818	87.3	341	89.7	0.193
	Used	160	11.8	2	4.9	119	12.7	39	10.3	
Family or friends	Not used	1194	87.9	37	90.2	829	88.5	328	86.3	0.522
	Used	164	12.1	4	9.8	108	11.5	52	13.7	
Internet and social media	Not used	994	73.2	29	70.7	695	74.2	270	71.1	0.462
	Used	364	26.8	12	29.3	242	25.8	110	28.9	
Maternal and Child Health Handbook	Not used	388	28.6	15	36.6	255	27.2	118	31.1	0.188
	Used	970	71.4	26	63.4	682	72.8	262	68.9	

Values are presented as n (%) within each maternal perception category (percentages are calculated by column). ^1^ *p* values were calculated using Fisher’s exact test. ^2^ See Table 2 for definitions of maternal perception and child body size categories.

**Table 4:** Association between maternal misperception of child body size and information-seeking behaviors and information sources.

		Maternal Misperception of Child Body Size ^1^															
		Overestimation (*n* = 41)								Underestimation (*n* = 380)							
		Model 1 ^2^				Model 2 ^3^				Model 1 ^2^				Model 2 ^3^			
		OR	95% CI		*p* Value	OR	95% CI		*p* Value	OR	95% CI		*p* Value	OR	95% CI		*p* Value
			Lower	Upper			Lower	Upper			Lower	Upper			Lower	Upper	
**Information Seeking Behavior**																	
Self-initiated information seeking about appropriate child weight/height	None	0.37	0.17	0.80	0.012	0.39	0.18	0.85	0.018	0.80	0.62	1.02	0.074	0.79	0.62	1.02	0.071
	Yes	1				1				1				1			
Opportunity to learn about appropriate weight/height	None	-	-	-	-	-	-	-	-	1.07	0.68	1.70	0.771	1.06	0.67	1.68	0.808
	Yes									1				1			
**Sources of information**																	
Healthcare providers	Not used	0.33	0.17	0.62	<0.001	0.39	0.20	0.74	0.004	0.84	0.64	1.11	0.216	0.87	0.66	1.15	0.339
	Used	1				1				1				1			
Public health professionals	Not used	0.83	0.43	1.58	0.567	0.99	0.51	1.91	0.971	1.12	0.87	1.44	0.391	1.16	0.90	1.51	0.250
	Used	1				1				1				1			
Childcare professionals	Not used	2.81	0.66	11.85	0.161	3.18	0.75	13.51	0.117	1.28	0.87	1.88	0.217	1.30	0.88	1.92	0.184
	Used	1				1				1				1			
Family or friends	Not used	1.19	0.42	3.43	0.741	1.33	0.46	3.85	0.595	0.82	0.58	1.18	0.286	0.85	0.59	1.22	0.369
	Used	1				1				1				1			
Internet and social media	Not used	0.88	0.44	1.75	0.708	1.21	0.59	2.48	0.611	0.84	0.65	1.10	0.210	0.91	0.68	1.21	0.509
	Used	1				1				1				1			
Maternal and Child Health Handbook	Not used	1.52	0.79	2.93	0.208	1.87	0.95	3.66	0.068	1.21	0.93	1.57	0.162	1.26	0.97	1.66	0.088
	Used	1				1				1				1			

OR—odds ratio. 95% CI, 95% confidence interval. ^1^ See Table 2 for definitions of maternal perception and child body size categories. ^2^ Model 1: Multinomial logistic regression analysis with accurate perception as the reference category. The independent variables included maternal information-seeking behaviors regarding child growth (1 = yes, 0 = none), opportunities to learn about the appropriate body size (1 = yes, 0 = none), and information sources regarding the appropriate body size (1 = used, 0 = not used). Models were adjusted for child age at the time of the survey, sex (1 = girl, 0 = boy), childcare attendance (1 = no, 0 = yes), and gestational age. Separate models were constructed for each information source. ^3^ Model 2: Additionally adjusted for maternal current employment status (1 = not employed, 0 = employed), maternal age, maternal BMI, and maternal HL score. In the analyses focusing on information sources, maternal information-seeking behaviors regarding child growth (1 = yes, 0 = none) were included. Separate models were constructed for each information source.

## Data Availability

The data sets used and/or analyzed during the current study are available from the corresponding author on reasonable request. The data are not publicly available due to privacy and ethical restrictions.

## Supplementary Materials

The following supporting information can be downloaded at https://www.mdpi.com/article/10.3390/children13030390/s1, Table S1: Associations of information-seeking behaviors and learning opportunities with maternal misperception of child body size (Models 1 and 2); Table S2: Associations between maternal misperception of child body size and information sources (Models 1 and 2).

## Abbreviations

The following abbreviations are used in this manuscript:

BMI	Body mass index
CI	Confidence interval
OR	Odds ratio
SD	Standard deviation
HL	Health literacy
MCHH	Maternal and Child Health Handbook
